# The accuracy of clinical neck staging for p16-positive and negative oropharyngeal cancer and its therapeutic implications

**DOI:** 10.1007/s00405-022-07430-7

**Published:** 2022-07-04

**Authors:** Timon Hussain, Kruthika Thangavelu, Cornelius Kürten, Lisa Galland, Benedikt Höing, Eric Deuss, Stefan Mattheis, Stephan Lang, Cornelius Deuschl, Michael Forsting, Nils Dörner

**Affiliations:** 1 Department of Otorhinolaryngology, Head and Neck Surgery, University Hospital Essen, University Duisburg-Essen, Hufelandstraße 55, 45147 Essen, Germany; 2 Department of Otorhinolaryngology, Head and Neck Surgery, University Hospital Marburg, Marburg, Germany; 3 Department of Radiology, University Hospital Essen, Essen, Germany

**Keywords:** Oropharyngeal squamous cell carcinoma, Neck metastases, Clinical staging, Extranodal extension, Therapy deintensification

## Abstract

**Purpose:**

Oropharyngeal squamous cell carcinoma (OPSCC) may be treated with primary surgery or primary (chemo)radiation. While surgery with concurrent neck dissection provides definitive pathological staging of the neck, non-surgical treatment relies on clinical staging for treatment planning. To assess the accuracy of clinical neck staging, we compared clinical to surgical staging after primary surgery in patients with p16-negative and p16-positive OPSCC.

**Methods:**

Retrospective analysis of clinical, pathological, and oncologic outcome data of patients with OPSCC treated with primary surgery and bilateral neck dissection. Clinical and pathological nodal status were compared for p16-negative and p16-positive patients. Patients with occult metastatic disease were analyzed in detail.

**Results:**

95 patients were included**.** 60.5% of p16-negative patients and 66.6% of p16-positive patients had pathologically confirmed metastatic neck disease. p16-positive patients had improved 24-month recurrence-free survival compared to p16-negative patients at 93.3% vs. 69.6%. Pathological N-status differed from clinical N-status in 36.8% of p16-negative patients vs. 31.6% of p16-positive patients. Occult metastatic disease was more common in p16-negative patients at 18.4% vs. 8.8% for p16-positive patients. Clinical detection sensitivity for extranodal extension was low overall; sensitivity was 27.3% and specificity was 91.6% for p16-negative patients vs. 61.5% and 80.0% for p16-positive patients, respectively.

**Conclusion:**

Our data show a considerable degree of inaccuracy of clinical neck staging results in all OPSCC patients which needs to be taken into consideration during therapy planning. For p16-positive patients, these findings warrant attention in the context of therapy deintensification to avoid undertreatment.

## Introduction

Oropharyngeal squamous cell carcinoma (OPSCC) comprises two fundamentally different biological cancer subtypes, HPV-associated OPSCC and non-HPV-associated OPSCC. Both may be treated either with primary surgery or primary radiation therapy, depending on disease characteristics, comorbidities, and patients’ preferences. OPSCC patients often present with neck metastases at the time of diagnosis [1], and reliable neck staging and subsequent adequate treatment are part of any therapeutic regimen. Compared to HPV-negative OPSCC, low-burden metastatic neck disease has a reduced negative prognostic impact in HPV-associated disease [2], as reflected in the current 8th edition of the American Joint Committee on Cancer (AJCC) staging manual. Compared to the 7th edition, clinical and pathological staging of the neck were modified substantially to distinguish between HPV-positive and HPV-negative OPSCC: HPV-positive patients with unilateral lymph node involvement may be considered stage I, depending on the size of the primary tumor, and patients with bilateral lymph node involvement may be considered stage II according to AJCC criteria; their HPV-negative counterparts would be considered stage III or IV, respectively. Nevertheless, advanced nodal status remains a significant negative prognostic factor in HPV-associated OPSCC [3, 4], and importantly, improved treatment responses in HPV-associated OPSCC patients compared to HPV-negative patients were achieved by patients undergoing treatment without HPV-adjusted deintensification. Accordingly, suspected or confirmed nodal metastasis currently mandates surgical or non-surgical neck treatment for all patients, regardless of HPV-status. If nodal metastasis is suspected according to clinical staging results or confirmed by pathology after neck dissection, a higher radiation dose is applied to the respective side of the neck, either as a primary treatment or during adjuvant treatment. Compared to non-surgical therapy, primary surgery with concurrent neck dissection for OPSCC has the inherent advantage of providing definitive pathological staging of the neck including additional risk factors such as extracapsular nodal extension (ENE), which then guide further treatment [5]. In cases of non-surgical primary therapy, physicians have to rely on the results of clinical neck staging for treatment planning, which may lead to an over- or under-treatment of the neck, depending on the accuracy of clinical staging. While favorable survival outcomes for HPV-positive OPSCC patients compared to their HPV-negative counterparts were initially established after primary radiation therapy, these findings have since been confirmed for patients undergoing primary surgery [1, 6, 7]. These results, combined with advances in transoral surgery, have led to a resurgence in primary surgical treatment for HPV-associated OPSCC with excellent disease control and good functional results [8–10]. Data from these surgically treated patients now allow for a comparison between clinical neck staging results and final pathology to determine the accuracy of clinical neck staging in OPSCC patients. In this study, we retrospectively analyzed data from OPSCC patients treated with primary surgery at our cancer center to determine the accuracy of pre-therapeutic clinical neck staging and guide future treatment decisions. p16-status served as a surrogate marker to differentiate between HPV-positive and HPV-negative patients.

## Methods

### Patients

We identified patients who underwent primary surgery for OPSCC at our institution between 2016 and 2020. Inclusion criteria were complete staging and initial treatment at our cancer center, no secondary malignancy at the time of diagnosis, availability of clinical as well as pathological staging data and the treatment recommendation of our interdisciplinary tumor board to perform primary surgery including bilateral neck dissection with curative intent. Routine staging included neck ultrasound, contrast-enhanced CT scan of the head and neck, contrast-enhanced CT scan of the thorax and ultrasound of the abdomen.

p16-status was routinely determined for all patients: immunohistochemistry was performed for p16INK4A, with a diagnostic threshold of diffuse nuclear and cytoplasmic staining in > 70% of tumor cells according to the European and College of American Pathologists’ guidelines.

All clinical and pathological parameters were recorded by chart review. This retrospective analysis was approved by the local ethics committee.

### Recurrence-free survival

24-month recurrence-free survival was determined for patients with sufficient follow-up and who met the inclusion criteria. Patients with incomplete follow-up records were excluded.

### Retrospective comparison of clinical and pathological neck staging

An experienced neuroradiologist blinded to pathological nodal status re-assessed all radiographic data including the CT scans as well as ultrasound images to determine clinical nodal status for all OPSCC patients, including clinical signs of extranodal extension. These data were compared to pathologic reports for all patients to identify differences between clinical and pathological nodal status. The predictive value of clinical neck staging was determined via receiver operator characteristic analysis and areas under the curve were calculated for both patient groups.

### Statistical analysis and data visualization

Part of the descriptive analysis, survival analysis including Kaplan–Meier curve and the Receiver Operating Characteristic curve were performed using Stata 14.0 software (Stata Corp. 2015). The Sankey diagrams were created using SankeyMATIC software (http://sankeymatic.com).

## Results

### Patients and treatment

95 patients who underwent primary surgery for OPSCC between 2016 and 2020 at our institution met the inclusion criteria. 60.0% (*n* = 57/95) of patients had p16-positive OPSCC, compared to 40.0% (*n* = 38/95) with p16-negative OPSCC. Patient and disease details are displayed in Table 1. While gender and age distributions were similar in both groups, p16-negative patients had more advanced, i.e. ≥ T3 primary tumors at the time of diagnosis (≥ T3 in 42.1% of p16-negative patients vs. 21.1% for p16-positive patients). In both groups, most patients had metastatic neck disease at the time of diagnosis (60.5% of p16-negative patients vs. 66.6% of p16-positive patients). Stage distribution according to the 8th edition AJCC criteria showed marked differences between groups with 70.2% of p16-positive patients being classified as stage I, compared to p16-negative patients where only 10.1% of patients were classified as stage I, and 47.4% of patients were stage IV.

**Table 1:** Overview over patients included in the study. Staging was based on the 8th edition American Joint Committee on Cancer (AJCC) staging manual for OPSCC

p16-positive patients (*n *= 57)		p16-negative patients (*n* = 38)	
Gender		Gender	
Male	75.4%	Male	66.8%
Female	24.6%	Female	34.2%
Age (avg.)	65.4 years	Age (avg.)	63.7 years
pT stage		pT stage	
1	29.8%	1	18.4%
2	49.1%	2	39.5%
3	19.3%	3	42.1%
4	1.8%	4	0%
pN stage		pN stage	
0	33.3%	0	39.5%
1	54.4%	1	13.2%
2	12.2%	2a	2.6%
3	0%	2b	18.4%
		2c	13.2%
AJCC stage		3	13.2%
		AJCC stage	
I	70.2%	I	10.1%
II	26.3%	II	18.4%
III	3.5%	III	23.7%
IV	0%	IV	47.4%

### Recurrence-free survival

**Fig. 1:**
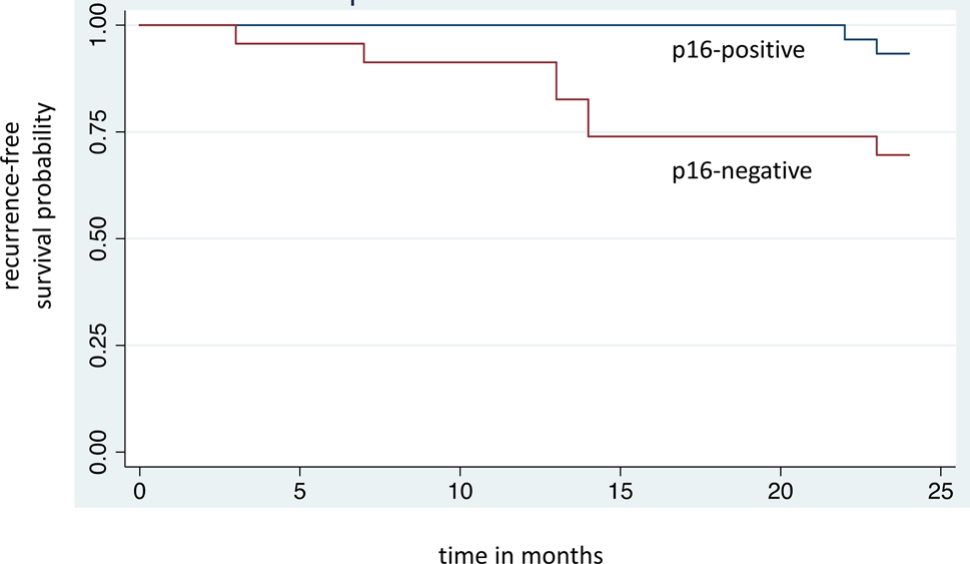
Kaplan–Meier curve showing improved 24-month recurrence-free survival for p16-positive patients (93.3%, *n* = 30) compared to p16-negative patients (69.6%, *n* = 23)

24-month recurrence-free survival for eligible patients is displayed in Kaplan–Meier curves as shown in Fig. 1. 93.3% (*n* = 28/30) of p16-positive patients were recurrence-free after 24 months, compared to 69.6% (*n* = 16/23) of p16-negative patients. Disease recurrence was confirmed by histology in all cases, accompanied by panendoscopy to exclude secondary malignancies. Of the two patients in the p16-positive group with disease recurrence, one patient had distant metastatic disease 23 months after primary treatment and one patient suffered from local recurrence 22 months after primary surgery and adjuvant radiation. The latter subsequently underwent salvage surgery with curative intent.

### Clinical vs. pathological neck staging

Clinical vs. pathological neck staging results were compared in the following three categories: no neck disease vs. unilateral neck disease vs. bilateral neck disease to determine the reliability of clinical neck staging compared to pathological staging.

In the p16-negative group, pathological N-status differed from clinical N-status in 36.8% (*n* = 14/38) of patients: 18.4% (*n* = 7/38) were up-staged, i.e. pathological N-status exceeded clinical N-status and 18.4% (*n* = 7/38) were down-staged, i.e. pathological N-status was lower than clinical N-status.

**Fig. 2:**
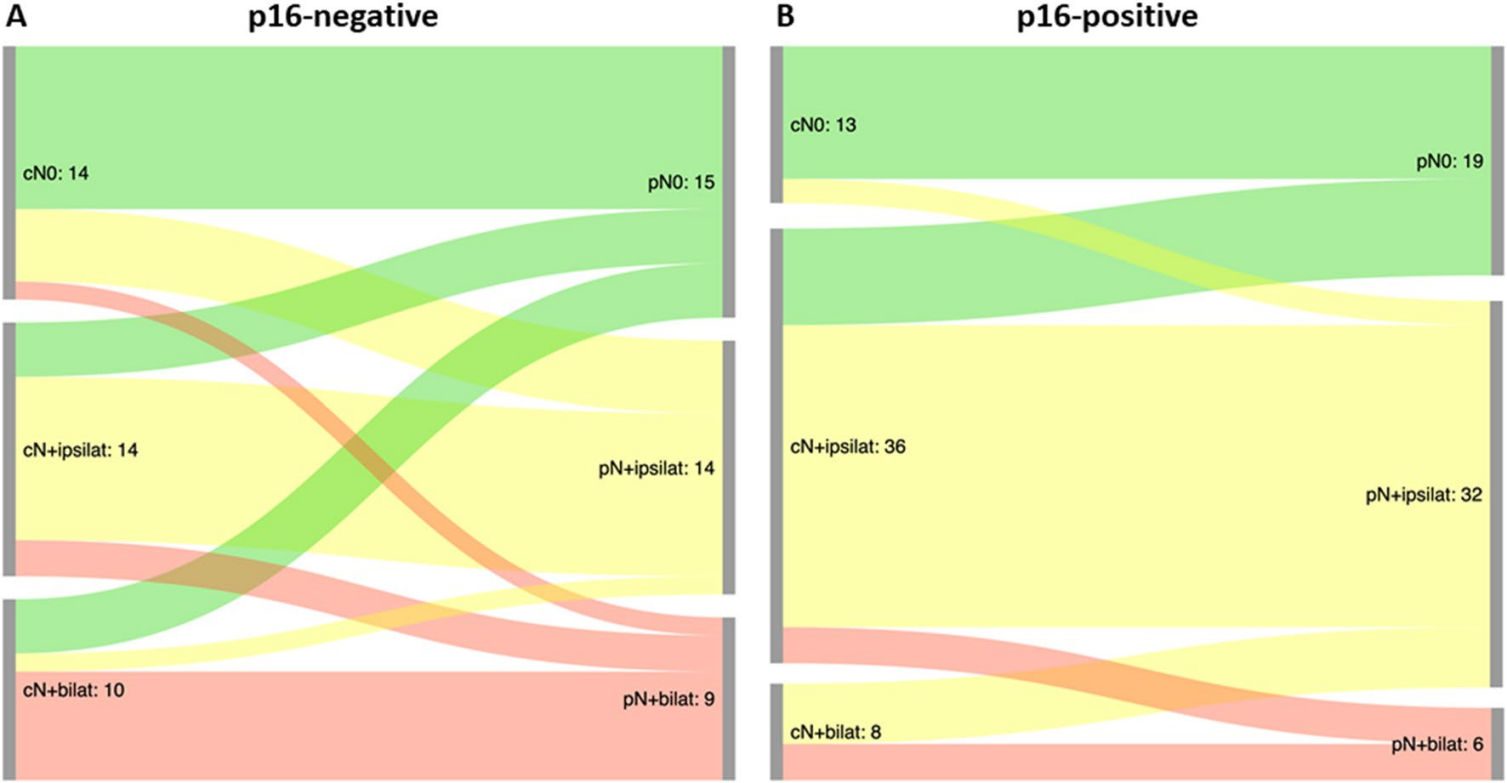
Sankey diagram visualizing the results of clinical vs. pathological staging for p16-negative (**a**) and p16-positive patients (**b**). 18.4% (*n* = 7/38) of p16-negative patients were up-staged (i.e. pN > cN) and 18.4% (*n* = 7/38) were down-staged (i.e. pN < cN) while 8.8% (*n* = 5/57) of p16-positive patients were up-staged and 22.8% (*n* = 13/57) were down-staged

In the p16-positive group, pathological N-status differed from clinical N-status in 31.6% (*n* = 18/57) of patients. 8.8% (*n* = 5/57) were up-staged and 22.8% (*n* = 13/57) were down-staged. The difference between groups was not statistically significant (95% CI − 13.4% to 24.3%; Chi squared 0.273; *p* value 0.6013). For better visualization, the results for both groups are displayed in a Sankey diagram in Fig. 2.

**Fig. 3:**
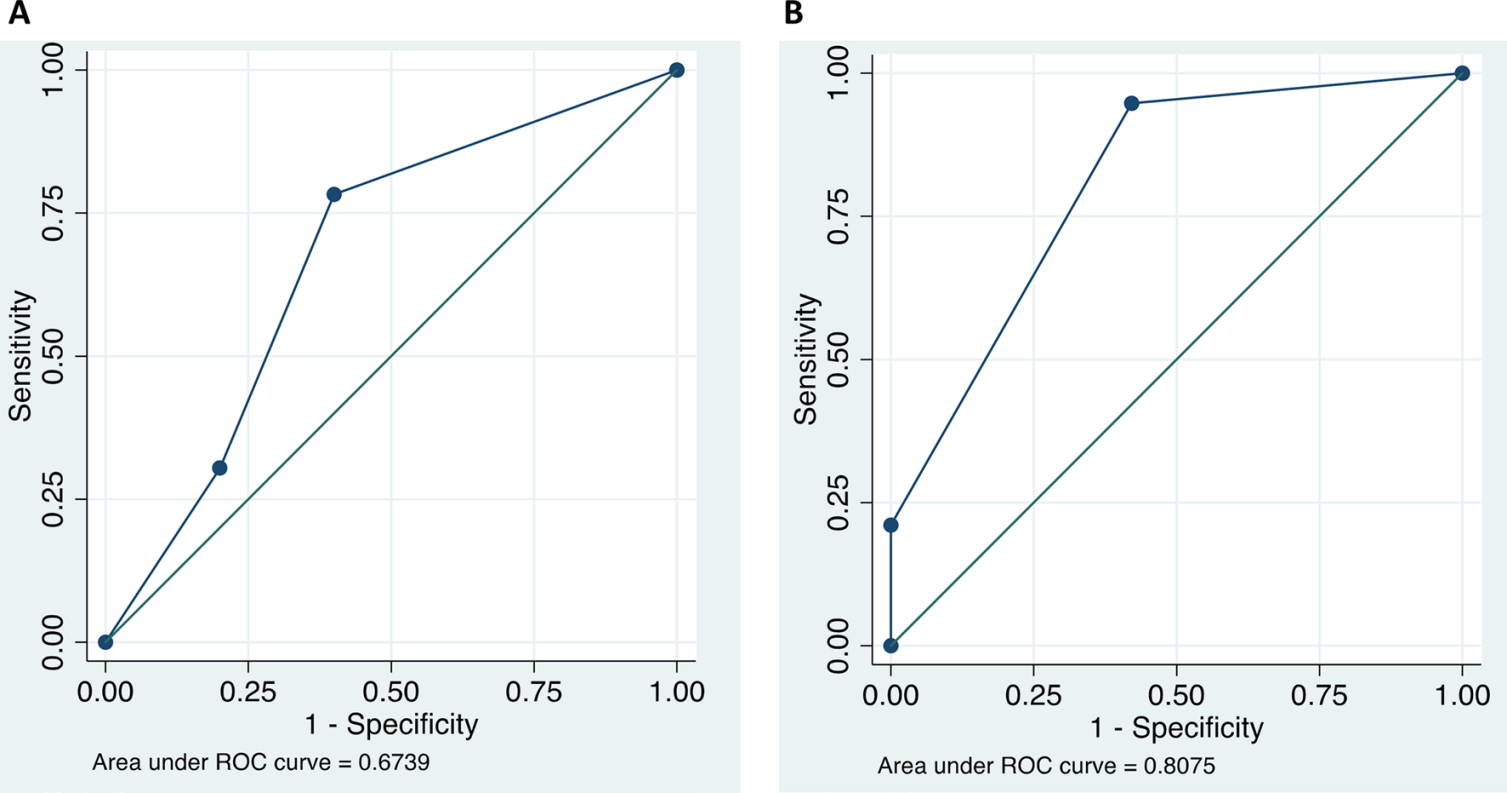
ROC analysis depicting the predictive value of clinical staging for metastatic neck disease in p16-negative OPSCC patients (**a**) and p16-positive OPSCC patients (**b**). AUC values were 0.67 and 0.81, respectively, indicating a higher predictive value of clinical staging for p16-positive patients

To determine the predictive value of clinical staging, we performed a Receiver Operating Characteristic (ROC) analysis. Results are shown in Fig. 3. Area under the curve (AUC) values were 0.67 for p16-negative patients and 0.81 for p16-positive patients, suggesting a greater reliability of clinical neck staging results in p16-positive patients compared to p16-negative patients.

A detailed overview of patients from both groups with occult metastatic disease is provided in Table 2. Occult metastases were more common in p16-negative patients compared to p16-positive patients and all clinically occult metastases were smaller than 1.5 cm in diameter.

**Table 2:** Detailed overview over patients with occult metastatic disease on either side of the neck. Occult metastatic neck lymph nodes were detected in both groups and across all tumor sizes and were more common in p16-negative patients

Patients with occult metastatic disease					
	Tumor localization	T-stage	Clinical N-stage	Pathological N-stage	Size of occult metastases
p16-positive					
Pat 1	Tonsil	1	N0	N1, ipsilateral metastases	1.0—1.5 cm
Pat 2	Base of tongue	2	N1, ipsilateral metastases	N1, bilateral metastases	1.0—1.5 cm
Pat 3	Tonsil	2	N1, ipsilateral metastases	N1, bilateral metastases	< 1 cm
Pat 4	Tonsil	3	N1, ipsilateral metastases	N2, bilateral metastases	< 1 cm
p16-negative					
Pat 1	Tonsil	1	N0	N1, ipsilateral metastases	< 5 mm
Pat 2	Tonsil	1	N0	N1, ipsilateral metastases	< 5 mm
Pat 3	Tonsil	2	N0	N2c, bilateral metastases	< 5 mm
Pat 4	Tonsil	2	N1, ipsilateral metastases	3b, bilateral metastases	1.0—1.5 cm
Pat 5	Base of tongue	2	N0	N2b, ipsilateral metastases	0.5—1 cm
Pat 6	Base of tongue	3	N1, ipsilateral metastases	N2c, bilateral metastases	0.5—1 cm
Pat 7	Base of tongue	3	N0	N2b, ipsilateral metastases	0.5—1 cm

### Extranodal extension: clinical vs. pathological assessment

Extranodal extension was detected during pathological work-up in 47.8% (*n* = 11/23) of p16-negative patients with neck metastases, and in 34.2% (*n* = 13/38) of p16-positive patients with neck disease. The difference was not statistically significant (95% CI − 11.2% to 36.5%; Chi squared 1.043; *p* value 0.3071). Detection accuracy for clinical signs of extranodal extension were as follows: For p16-negative patients, detection sensitivity was 27.3% and specificity was 91.6%; For p16-positive patients, detection sensitivity was 61.5% and specificity was 80.0%.

## Discussion

Our results show a considerable discrepancy between clinical neck staging results and final pathological findings after bilateral neck dissection in p16-positive and p16-negative patients. To account for clinical relevance, and under the assumption that any clinical sign of neck disease would warrant surgical treatment or full-dose radiation, we chose to compare three categories without further sub-classification: no neck disease vs. unilateral neck disease vs. bilateral neck disease. Under these criteria, discrepancy rates between clinical and pathological neck status were more than 30% for both p16-positive and p16-negative patients, including both up-staged and down-staged patients in both groups. The share of pathologically up-staged patients, i.e. with pathological proof of clinically occult metastatic disease, while not yielding statistical significance in our patient sample, tended to be lower for p16-positive patients compared to their p16-negative counterparts. A potential explanation for a lower rate of occult metastases among p16-positive patients may be the oftentimes cystic nature and larger size of HPV-associated metastatic lymph nodes [11–13]. Nevertheless, occult metastatic disease was detected in nearly 10% of p16-positive patients across all stages. In both groups, primary tumor size did not seem to correlate with the occurrence of occult metastases. In both groups, occult metastases were less than 1.5 cm in size, and could not be reliably distinguished from reactive changes in regional lymph nodes upon CT imaging. Patients included in this study all underwent bilateral neck dissection and adjuvant therapy according to the pathological results, therefore, occult metastatic disease did not lead to undertreatment; however, had the same patient collective been treated non-surgically, 18.8% of p16-negative and 8.8% of p16-positive patients would have received treatment with an underlying underestimation of their metastatic disease burden.

For HPV-positive patients, the reliability of clinical staging warrants special attention in the context of potential therapy de-escalation attempts. The markedly better treatment response of HPV-associated OPSCC compared to HPV-negative patients has led to therapy deintensification efforts with multiple ongoing prospective studies [14]. Our results, which confirm superior disease-free survival for p16-positive vs. p16-negative patients fall in line with multiple retrospective studies from recent years showing excellent disease control in HPV-positive patients after primary surgery—notably with patients undergoing un-adjusted treatment regimens, i.e. regular dose adjuvant therapy where warranted [1, 15]. Preliminary results of deintensification trials for adjuvant treatment after primary surgery have been promising, however, deintensification was carefully adjusted to pathological results, for example excluding patients with high burden metastatic neck disease, extranodal extension or other potential risk factors which were determined by pathology [16]. Deintensification strategies for primary *non*-surgical treatment are currently under investigation including the omission of chemotherapy or the reduction of radiation dose to the tumor region, or the neck [17]. Here, treatment planning relies solely on clinical staging. The degree of inaccuracy of clinical staging as shown by our analysis, particularly regarding occult metastatic disease, has to be taken into consideration to avoid undertreatment, hereby potentially compromising the otherwise excellent prognosis of HPV-positive OPSCC patients.

It is worth mentioning that while undertreatment may be more consequential for the patients’ oncologic outcome, potential overtreatment should be avoided where possible to prevent adverse effects on patients’ quality of life. In our patient collective, the degree of neck metastasis was overestimated in about 20% of patients in both groups during clinical staging. During primary radiation therapy, this would have increased radiation doses to the respective side of the neck, hereby potentially adding morbidity, particularly regarding swallowing function [18]. In patients with surgically resectable tumors, neck dissection provides definitive pathological staging, enabling highly accurate postoperative adjuvant treatment. Of course, for patients where both primary surgery and primary radiation are viable therapeutic options, the degree of potential surgical morbidity must be considered and weighed against the risk of potentially applying unwarranted radiation doses. An awareness of the pitfalls of clinical staging, as shown in our data, should aid physicians during treatment planning and patient counseling.

**Fig. 4:**
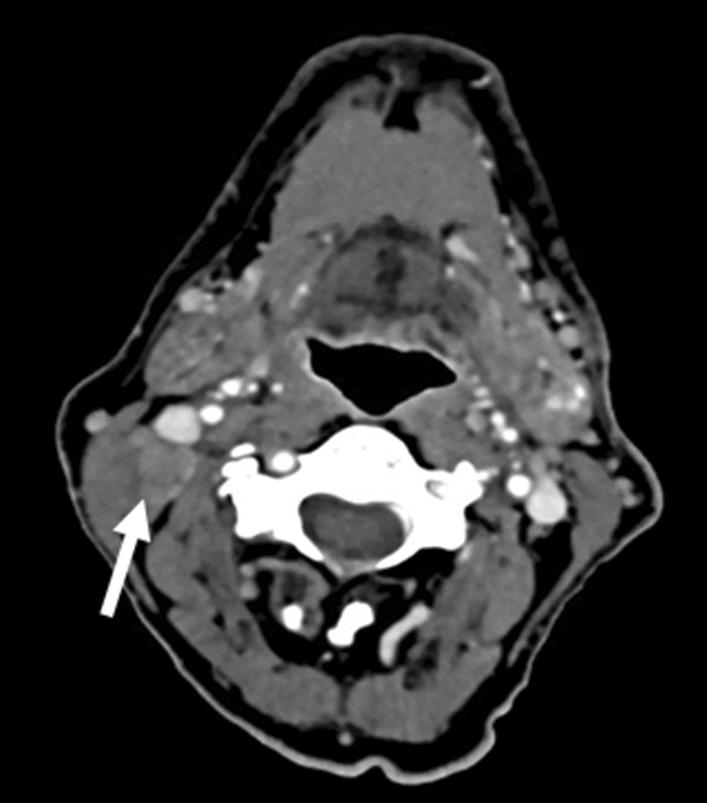
CT scan of a patient with a base of tongue OPSCC with right cervical lymph node metastasis with clinical features of extranodal extension which were confirmed by pathology (white arrow)

Aside from determining overall nodal status, pathological staging facilitates the assessment of additional potential nodal risk factors, such as extracapsular nodal extension (ENE). In this study, we determined the accuracy of clinical detection of ENE in our patient collective, revealing low levels of detection accuracy, particularly sensitivity. Cases with clinically obvious extracapsular extension, as shown in Fig. 4, were rare, especially in the group of p16-negative patients where sensitivity was less than 30%. ENE is a strong negative prognostic factor in HPV-negative OPSCC [19]. In HPV-associated OPSCC, the prognostic role of ENE has lately been a topic of debate. While suggested to have little influence on survival by some [20, 21], others have recently suggested a significant negative impact in HPV-associated OPSCC [22, 23], and recommend taking ENE into consideration when planning treatment. In our patients, ENE tended to be less common in p16-positive disease compared to p16-negative disease but nevertheless present in more than 30% of patients with neck metastases. Augmented imaging technologies, such as PET/CT have also been shown to poorly predict ENE in OPSCC [24], therefore, surgery is currently the only reliable detection method.

In conclusion, our data reveal a considerable degree of inaccuracy for the clinical detection of lymph node metastases and associated adverse features in OPSCC patients. Our single-center approach allowed for an in-depth analysis of patient and disease characteristics, such as the size of occult metastases. At the same time, it also limited the sample size and differences between p16-positive and p16-negative patients need to be confirmed in larger patient samples. Occult metastatic disease was detected in both groups yet tended to occur less frequently in HPV-positive patients. These findings must be considered during therapy planning and patient counseling for all OPSCC patients, particularly where both primary surgery and primary radiation are feasible. Particular attention is warranted during therapy de-escalation attempts for HPV-associated disease without primary surgery which would provide definitive pathological staging of the neck to avoid undertreatment of patients with an otherwise excellent prognosis.

## Data Availability

N/A.

## Code availability

N/A.
